# Dissecting the Environmental Consequences of *Bacillus thuringiensis* Application for Natural Ecosystems †

**DOI:** 10.3390/toxins13050355

**Published:** 2021-05-16

**Authors:** Maria E. Belousova, Yury V. Malovichko, Anton E. Shikov, Anton A. Nizhnikov, Kirill S. Antonets

**Affiliations:** 1 Laboratory for Proteomics of Supra-Organismal Systems, All-Russia Research Institute for Agricultural Microbiology (ARRIAM), 196608 St. Petersburg, Russia; m.belousova@arriam.ru (M.E.B.); yu.malovichko@arriam.ru (Y.V.M.); a.shikov@arriam.ru (A.E.S.); a.nizhnikov@arriam.ru (A.A.N.); 2 Faculty of Biology, St. Petersburg State University, 199034 St. Petersburg, Russia

**Keywords:** *Bacillus thuringiensis*, ecology, entomophages, pathogen, biopesticide

## Abstract

*Bacillus thuringiensis* (*Bt*), a natural pathogen of different invertebrates, primarily insects, is widely used as a biological control agent. While *Bt*-based preparations are claimed to be safe for non-target organisms due to the immense host specificity of the bacterium, the growing evidence witnesses the distant consequences of their application for natural communities. For instance, upon introduction to soil habitats, *Bt* strains can affect indigenous microorganisms, such as bacteria and fungi, and further establish complex relationships with local plants, ranging from a mostly beneficial demeanor, to pathogenesis-like plant colonization. By exerting a direct effect on target insects, *Bt* can indirectly affect other organisms in the food chain. Furthermore, they can also exert an off-target activity on various soil and terrestrial invertebrates, and the frequent acquisition of virulence factors unrelated to major insecticidal toxins can extend the *Bt* host range to vertebrates, including humans. Even in the absence of direct detrimental effects, the exposure to *Bt* treatment may affect non-target organisms by reducing prey base and its nutritional value, resulting in delayed alleviation of their viability. The immense phenotypic plasticity of *Bt* strains, coupled with the complexity of ecological relationships they can engage in, indicates that further assessment of future *Bt*-based pesticides’ safety should consider multiple levels of ecosystem organization and extend to a wide variety of their inhabitants.

## 1. Introduction

*Bacillus thuringiensis* Berliner (*Bt*) is a Gram-positive, aerobic, spore-forming, and endotoxin-producing bacterium [1], firstly mentioned by Shigetane Ishiwata back in 1902 and described as *Bacillus sotto* [2]. Later in 1915, the bacterium was rediscovered by Ernst Berliner, who isolated it from a Mediterranean flour moth, *Ephestia kuehniella,* in the German province of Thuringia, from which it received its commonly used species name, *Bacillus thuringiensis* [3]. Due to its pathogenicity against insects, shortly after the discovery, the first suggestions on the possibility of using *Bt* in pest control were made. In 1927, Mattes carried out field tests of a strain re-isolated from *E. kuehniella* on a European corn moth [4]. *Bt* inclusions produced in the stationary phase were called “parasporal crystals” by Christopher Hannay in 1953. Later, these inclusions were shown to comprise proteins with insecticidal properties [5,6]. Nowadays, the production of these toxins is considered a key feature of *Bt*, allowing it to become an efficient pest control agent.

*Bt* belongs to *Bacillus cereus* sensu lato (or the *Bc* group), a taxonomic unit of volatile species content [7,8]. Initially, the *Bc* group comprised three relatively distinct species, namely, *B. anthracis*, *B. cereus,* and *B. thuringiensis*, which were characterized based on clinically and agriculturally important phenotypic traits [9]. For instance, *B. anthracis* was described as an anthrax-causing agent, and *B. cereus* is an opportunistic pathogen [9]. In its turn, *Bt* is characterized by parasporal crystalliferous toxins with insecticidal activity [9]. However, since then, the species content of the *Bc* group has substantially expanded. By now, different authors distinguish 6 [9], 8 [10], or 11 [11] species. The advances of genome-wise phylogeny have allowed for the identification of 20 [11] and even 37 putative genomospecies [7]. Alternatively, prior to the aforementioned phylogenetic studies, the *Bc* group had been suggested to be perceived as a single species with immensely diversified and adaptable phenotypes [1,12]. An appropriate trade-off between practical usability which also accounted for evolutionary relations was proposed by Carroll et al. (2020) [8]. Based on average nucleotide identity (ANI)-thresholds coupled with phenotypic traits, *Bacillus thuringiensis* was proposed to stand for biovar Thuringiensis presented in *B. mosaicus* and *B. cereus* sensu stricto genomospecies. As the taxonomic subtleties are beyond the present article’s scope, here we would use the typical term *Bacillus thuringiensis.*

Superficially, both the toxicity and specificity of *Bt* can be attributed to its proteinaceous toxins, which either constitute the parasporal crystals (Cry and Cyt superfamilies) or are excreted once the bacteria infiltrate the host’s hemolymph (Vip and Sip superfamilies) [13,14]. However, the actual list of virulence factors affecting *Bt*’s pathogenicity involves numerous other moieties, including both proteins and low weight substances [15]. Two other major groups of virulence-enhancing enzymes include phosphoinositide-specific phospholipases C (PLC), chitinases, and metalloproteases. While the activity of chitinases and PLCs is mainly confined to the eradication of peritrophic structures (reviewed in [16]), metalloproteases demonstrate a more versatile range of functions. Most of them degrade either peritrophic membranes [17] or basal lamina [18,19,20]; however, members of the InhA family also mitigate the host’s cellular [21] and humoral [22] immunity, and CalY process biofilm matrix proteins [23] and regulate the activity of other proteases and Cry toxins [24,25,26]. PLCs were found to serve as a virulence enhancer in all major species of *Bacillus cereus* sensu lato [27,28]. Apart from the effector proteins, the flagellar apparatus is also crucial for an effective *Bt* infection. Smaller moieties comprise nucleoside-mimicking β-exotoxins, which inhibit RNA polymerase activity [29], *trans*-aconitic acid, a potent inhibitor of the tricarboxylic acid cycle [30], and zwittermycin A, an aminopolyol antibiotic [31]. The latter ameliorates *Bt* pathogenicity by debilitating the host’s gut microflora [32], giving way to the thought that other antimicrobial moieties produced by *Bt* may impact its virulent features [33].

Biological insecticides based on *Bacillus thuringiensis* (*Bt*) are widely represented and utilized worldwide for plant protection and mosquito control. The first *Bt*-pesticide (Sporeine) was commercialized in France in 1938 and applied to control lepidopteran pests [4]. At present, more than 400 *Bt*-products have been developed, derived from a small number of homologated progenitor strains. Most of these strains are explicitly attributed to serovars as determined by serological properties revealed by agglutination assays. This well-established classification, however, has been proven to be contradictory based on genomic and phenotypic data; nonetheless, it still finds wide application in *Bt* studies [34,35]. Biopesticides derived from *B. thuringiensis* var. *thuringiensis* show activity against Lepidoptera and Coleoptera pests; however, there are insecticidal preparations implementing other serovars, such as *kurstaki*, *aizawai*, *san diego*, and *tenebrionis.* [36,37,38]. *Bt* insecticides usually contain Cry toxins and spores as active ingredients [37] but preparations consisted of encapsulated Cry toxins have also been developed [39]. Exotoxin-producing strains are prohibited for registration as a control agent in Europe, the US, and Canada, due to their toxicity to mammals [13,40,41]. However, in some countries, exotoxin-containing preparations are still used, such as preparation Bitoxybacillin (Russia), based on the *Bt* var. *thuringiensis* strain capable of producing both exotoxin and Cry toxins [42,43]. Spores contained in *Bt*-pesticides are also intensively discussed in the context of stability in the environment and the ability to accumulate in the soil [44,45,46,47,48] and non-target organisms [49]. Even though spores can enhance insecticidal activity by contributing to the development of septicemia in target organisms, manufacturers aim to develop spore-free formulations. Nevertheless, many researchers support *Bt* application as a safer alternative to chemical insecticides, especially in organic farming [37].

**Figure 1:**
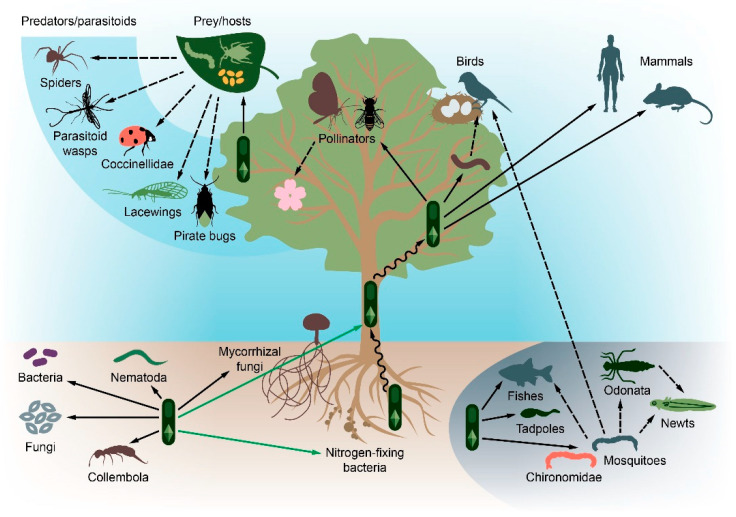
The effect of *Bt* on organisms within the natural ecosystems. Black arrows indicate possible detrimental effects; green arrows denote possible beneficial effects. Solid arrows denote direct effects; dotted arrows stand for indirect effects. Black wavy arrows indicate *Bt* transfer through plant tissues.

The action of *Bt* on target objects is considered to be specific due to Cry toxins’ mode of action and special conditions for protoxin activation in the intestines of target insects [50]. The general safety of the Bt spores and crystals usage, particularly compared with chemical insecticides, are reviewed in previous papers [51,52]. Nonetheless, the possible side effects caused by the introduction of Bt on the non-target organisms and the ecosystems, on the whole, are articulated infrequently. Because they are complex systems comprising hundreds of different species, ecosystems can be studied at different organization levels and food chain stages; namely, the microscopic level with all microbial communities, the level of small animals such as worms and insects, the level of large animals, mammals and birds, and the ecosystem overall. Here, we discuss accumulated evidence on *Bt*’s impact at different ecological levels, paying particular attention to the emergence of systemic effects, highlighting inadequately studied issues, and providing a comprehensive ecotope-wise scheme summarizing the direct and indirect *Bt* application effects (Figure 1).

## 2. Soil Microbial Communities

Microbial communities are a crucial part of any ecosystem, predominantly found in field and forest soils. Soil microorganisms form a remarkably diverse and numerous ecological group, comprised of bacteria, fungi, soil protozoa, and soil algae, in descending order [53]. These microorganisms take part in the decomposition of organic matter, nitrogen fixation, nutrients transformation, and detoxification of toxic substances. They also facilitate whole fertility and productivity when interacting with other soil components [53]. *Bt*’s ecological role in the soil is extensively studied in terms of interactions with other microorganisms, the impact of physical and chemical soil characteristics on the life cycle, and the dispersion path.

*Bt* is capable of colonizing and surviving in various ecotopes, such as water, plants, stored cereals, dead insects, and vertebrate feces [54]. However, due to the soil being the most frequent source of isolation, many researchers perceive *Bt* as a natural soil inhabitant. So far, *Bt* strains have been isolated from the soils having not experienced exposure to *Bt*-based products [55,56,57,58,59]. The total soil *Bt* population is thus constituted by natural soilborne strains, strains introduced with the application of pesticide, and strains transferred from other local biotopes, such as phyllosphere, insect cadavers, or animal feces [60,61,62,63].

Spores and proteins included in preparations behave differently while entering the soil. In most cases, the crystal proteins are inactivated within days in the phyllosphere and soil [64]. The fastest toxin inactivation rates were recorded during the first 45 days upon application, and less than 25% of the initial bioactivity remained after 120 days [65,66]. However, protein toxins can also bind clay particles in the soil, which reserves insecticidal activity [67,68]. Endospores can remain viable in the environment for more than 2 years [69]. It was demonstrated that *Bt* spores tended to persist longer in substrates containing organic matter compared to sand-only substrates [70]. It should be noted that spores rarely germinate in soil environments. Several properties of soil substrates, such as moisture, pH, and the presence of other microorganisms, were proved to have little to no effect on spore survival and germination [71]. However, *B. thuringiensis* germination and propagation in the soil is limited by nutrient availability, and vegetative cells tend to die quickly in non-sterile soil [72,73]. Spore recycling is probably related to the presence of target insects [74]. The ability to proliferate and multiply was shown for strain *Bt* serovar *israelensis* IPS82, and the authors consider that other soil microorganisms can release specific components that serve as nutrients enabling the development of *Bt* spores [75]. Contrariwise, the indigenous soil bacteria *Pseudomonas fluorescens* secretes toxic compounds affecting the development of *Bt*’s vegetative cells, which induces sporulation and hampers multiplying [75].

The production of antimicrobial agents may, in the long run, drastically affect the composition of the microbial community. The activity of thuricins, the *Bt*-produced antimicrobial peptides, was tested on the closely related bacterial species, such as various *Bt* serovars and non-*Bt* bacteria of the *Bc* group [76,77,78,79], as well as for more distant Gram-positive bacteria [78,80,81,82,83,84,85]. In comparison, the effect of *Bt* thuricins on Gram-negative bacteria is less pronounced. Ugras et al. [86] described Thuricin Bn1 produced by *Bt* var. *kurstaki,* which is active against Gram-negative plant pathogenic bacteria residing in soil *(Paucimonas lemoignei, Pseudomonas syringae, P. savastanoi*). Most likely, *Bt* thuricin interacts with the cell membrane with subsequent provoking of the transmembrane potential dispersion [82]. The ability to produce bacteriocins can affect populations of indigenous soil microorganisms. Zhang et al. studied the dynamics in the ratio of the species composition of natural soil bacteria after treatment of *Bt* in the rhizosphere of pepper plants [87]. Members of Firmicutes phylum, including those of genera *Bacillus*, *Clostridium*, *Desulfotomaculum*, *Enterococcus*, and *Paenibacillus*, prevailed on non-treated sites, while for *Bt*-treated sites, Gammaproteobacteria were the most abundant. This observation could be explained by the direct competition for nutrients or the *Bt* toxins impact on some bacteria [87]. Convincing illustration of bacterial interspecies competition has been demonstrated by Houry et al. [88], when the lysostaphin-producing *Bt* strain was able to replace native *Staphylococcus aureus* from soil biofilm population.

Some of the soil bacteria can participate in beneficial symbioses with plants. A prominent example of such interactions is represented by nitrogen-fixing nodule bacteria, the symbionts of legumes [89]. It has been shown that Bt’s co-inoculation with the symbiotic bacterium *Rhizobium leguminosarum* can stimulate the formation of pea and lentil nodules; there is also no inhibitory effect of *Bt* on *Rh. leguminosarum* [90]. No effect on heterotrophic bacterial populations was found, but *Bt* inoculation increased nodule formation and plant growth. However, chitinase produced by *Bt* subsp. *pakistani* HD 395 can degrade the Nod factor of saprophytic nitrogen-fixing soybean symbiont *Bradyrhizobium japonicum*, which can interfere with signaling nodule formation [91].

A number of chitinases produced by *Bt* strains exhibit antifungal effects [92,93]. Djenane et al. [92] have shown the antifungal activity of almost all *Bt* isolates found in the rhizosphere. Antifungal action of *Bt* on *Fusarium solani, F. oxysporum, F. proliferatum, Colletotrichum sp., Rhizoctonia cerealis, Rh. solani, Verticillium dahliae,* and *Bipolaris papendorfii* [16,93,94] was reported. It is likely that these activities may impair the arbuscular mycorrhizal fungi. While pure δ-endotoxin solution did not adversely affect sorghum-associated arbuscular mycorrhiza [73,95], Cry-producing and non-producing strains can inhibit colonization of mycorrhiza when compared with uninoculated plants [73].

Thus, soil serves as a reservoir for *Bt*, which are represented by both indigenous populations and strains introduced with the application of the biopesticides. *Bt* presence in the soil is usually associated with spores that can germinate under special conditions rich in organic substrates, found in some plants’ rhizosphere or the intestines of soil invertebrates. However, vegetative cells are inhibited by competition with other microorganisms or adverse environmental conditions. Some limitations of *Bt* to the development of the rhizosphere and soil, such as the inability to colonize roots effectively compared to other rhizosphere bacteria and growth restrictions in environments with insufficient amounts of nutrients, do not allow us to consider these niches to be preferred. Nevertheless, they could be essential as a means to transfer to other niches and as a preserving reservoir. Colonizing the rhizosphere or persisting in soil, the bacterium becomes available for ingestion by invertebrates feeding on roots, plant debris, and soil organic matter (Figure 1). Although the soil is not a preferable environment, *Bt* can affect soil communities and stimulate plant growth.

## 3. Plants

Plant-associated habitats, such as rhizosphere and phyllosphere, shelter diverse microbial communities. The effective colonization of the rhizosphere relies on the availability of organic matters, such as carbohydrates and amino acids, in which plant root exudates are enriched [54]. The presence of *Bt* either in the rhizosphere or as an endophytic bacterium was shown in cotton, soybean, maize, sugarcane, and cabbage [96,97], and in the rhizosphere of dandelion and quackgrass, *Bt* vegetative cells were found to constitute a high proportion of the associated communities [98,99].

Spores and parasporal bodies may not only penetrate the plant’s root tissues, but also further transfer to its terrestrial parts, as shown in soybeans [100]. What is noteworthy, *Bt* crystals retained their insecticidal activity after isolation from terrestrial soybean tissues. For successive translocation, *Bt* vegetative cells can produce the enzymes degrading components of plant cell walls in a manner similar to that of plant pathogens. However, in cabbage, *Bt* was demonstrated to colonize root tissues exclusively in the form of spores, with no vegetative cells having been registered [96]. An alternative to chemical degradation of the root tissues, the penetration of *Bt* was proposed to occur through physical ruptures in the roots and through natural openings associated with the secondary root formation [96].

By colonizing terrestrial plant tissues, the indigenous *Bt* populations found in the areas unexposed to *Bt*-based pesticides treatment may serve as natural plant protection agents [59]. In the experimental setup, leaves taken from the naturally colonized plants demonstrated toxicity when fed to the lepidopterans *Spodoptera frugiperda* and *Plutella xylostella*, associated with cotton and cabbage, respectively. The ability of *Bt* to colonize the roots is considered to be lower than that of other rhizosphere bacteria, such as *Pseudomonas* species; however, *Bt* was shown to persist in roots even in the presence of such competitors [101]. The ability to successfully colonize plant tissues is likely to correlate with the presence of specific genes, such as those encoding indolpyruvate decarboxylase, which enables bacteria to influence auxin production in plants [101].

With the ability to produce antifungal [102,103] and antimicrobial [104] agents, *Bt* may exert plant growth promotion by means of mitigating pathogens’ activity. Alternatively, rhizosphere *Bt* can directly promote plant development [105]. This beneficial trait refers to the ability of some *Bt* strains to produce phytohormones, such as auxins, or to control ethylene balance in plants [100,105,106]. Some strains can enhance plant uptake of iron and phosphorus [106] or increase plant resistance to abiotic stresses [107].

Thus, *Bt* exerts a multidirectional effect on plants. *Bt* can significantly enhance the nodulating activity of nitrogen-fixing bacteria and suppress mycorrhizal fungi growth, with the former phenomenon being unequivocally positive and the latter one being highly detrimental for plant growth and maintenance, respectively. However, the same mechanisms that mediate mycorrhiza growth suppression are involved in deteriorating the pathogenic fungi activity, which again brings benefits to plants, with which resident *Bt* strains are associated (Figure 1). Apart from these activities, *Bt* can also promote plant growth by excreting siderophores and phytohormone precursors. Arguably the most crucial point of mutualistic integration between plants and *Bt* comes from its ability to penetrate roots and migrate through plant tissues to the terrestrial organs maintaining insecticidal activity. By utilizing this strategy, the soilborne *Bt* strains, both indigenous and introduced with the biopesticides application, may serve as natural insecticides of prolonged conservation and activity. The apparent benefit of colonizing terrestrial plant tissues lies in the expanded range of dissemination and the elevated probability of encountering the target insect host. However, the complexity of these interactions brings in the risk of the additional off-target effects accompanying *Bt* preparations application, including those exerted on beneficial organisms.

## 4. Invertebrates

### 4.1. Soil Invertebrates

Soil invertebrates constitute an important element of the ecosystem. Invertebrates are involved in numerous processes occurring in the soil, such as infiltration and storage of water in the soil pore system, decomposition, and association of soil particles, pedogenesis, and dispersal of microorganisms [108]. With respect to their size, soil invertebrates are divided into three groups [108]. The smallest soil invertebrates, or microfauna, are represented by nematodes; mesofauna, or middle-sized animals, including microarthropods and *Enchitraeidae*; finally, macrofauna encompasses soil mollusks, crustaceans, and *Lumbricidae* species. As they are ubiquitously represented in the soil, these organisms can get in contact with both resident *Bt* populations and those introduced upon *Bt* insecticide treatment. Indeed, *Bt* was isolated from earthworms [99], nematodes [109,110], and soil crustaceans [111].

Soil nematodes diverge into several ecological groups regarding their feeding behavior. For instance, herbivorous nematodes include both ectoparasites feeding on the root surface and endoparasites capable of penetrating plant roots residing in root tissues. Bacterivores and fungivores are free-living nematodes which feed on bacteria and fungi, respectively, and play an important role in the decomposition of organic matter. Through their feeding, these nematodes return minerals and other nutrients to the soil, making them accessible for plant roots [112]. Predatory nematodes feed on other soil invertebrates of animals of comparable size. Finally, omnivorous nematodes can feed on different types of substrates [112]. Due to their ubiquity, soil nematodes pose as perfect hosts for soilborne *Bt;* consequently, some *Bt* strains produce nematicidal toxins [110]. The susceptibility to Cry5B, Cry14A, Cry21A, Cry6A toxins was demonstrated for free-living *Caenorhabditis elegans, Pristionchus pacificus, Panagrellus redivivus, Panagrellus redivivus, Distolabrellus veechi, Acrobeloides,* and parasitic *Nippostrongylus brasiliensis* nematodes, by using intoxication, developmental, fertility, and gut morphology assays [113]. Interestingly, bacterivorous soil nematode can demonstrate a defensive strategy against pathogens, such as physical evasion from pathogens and reduced oral absorption. No toxicity was shown for *C. elegans* when exposed to *Bt* var. *kurstaki* and Cry1Ac protein [113]. In addition to nematicidal Cry toxins, some *Bt* strains produce other nematicidal virulence factors, such as metalloproteases and *trans*-aconitic acid (TAA) [15,19,20,30]. Metalloproteases serve mostly for host penetration and further advance of septicemia; in addition, some metalloproteases may synergize with nematicidal Cry toxins or condition their activation [19,20]. In turn, TAA was proved to be active against the plant-parasitic nematode *Meloidogyne incognita* [30]. The nematicidal activity of *Bt* is used for controlling the plant-pathogenic nematodes in agriculture [114,115]. So far, *Bt* toxicity was demonstrated for such plant pathogenic nematode as *Meloidogyne hapla*, *Pratylenchus scribneri*, *Tylenchorhynchus* sp., *Ditylenchus destructor*, and *Aphelenchoides* sp. [116].

As a side effect of their nematicidal activity, *Bt* might infect entomopathogenic nematodes, which are considered to be promising agents for soilborne pest control [117]. Pesticides based on *Bt* and entomopathogenic nematodes are often used simultaneously, and most researchers consider these two plant protection agents to be fully compatible, with their synergistic effect having been described [118,119]. *Bt* sprayed on foliage does not affect the effectiveness of entomopathogenic nematodes; however, the mortality of nematodes (*Steinernema carpocapsae* and *Heterorhabditis bacteriophora*) is noted when they are contained in a bacterial suspension [118].

Earthworms account for most of the soil macrofauna biomass in practically all communities. They are involved in maintaining the nutrient cycle in the soil and can increase the activity of beneficial soil microorganisms due to mucus secretion, which leads to an increase in the nutrient (namely, nitrogen, phosphorus, potassium, and calcium) availability to plants [120]. *Bt* was found in the gut of earthworms, although the possibility of increasing the number of vegetative cells in the gut has not been shown [99]. Nonetheless, spores may germinate in the intestinal environment, thus conditioning the earthworms’ contribution to the dissemination of *Bt* in the soil habitat. Experiments have shown that *Bt* is harmless to earthworms when applied at normal field rates [121], while the extremely high doses of bacteria cause fatal septicemia [122].

Data on the side effects of *Bt* on other soil invertebrates are limited. The application of the commercial insecticide Dipel ES^®^ in a deciduous forest setup did not affect major soil fauna groups, such as earthworms, enchytraeids, oribatids, gamasids, and collembolans, but reduced the local dominance rank of mite *Veigaia nemorensis* [123]. Some toxicity of the purified δ-endotoxin produced by the Antarctic *Bt* strain was shown for two collembolan species, namely *Folsomia candida* and *Seira domestica* [124]. However, the observed toxicity was negligible, and the authors believe that it is unlikely to cause serious consequences in natural conditions [124].

### 4.2. Arthropods

Terrestrial crustaceans can internalize *Bt* spores by swallowing contaminated soil particles and feces. Swiecicka [111] considers *Bt* a true isopod commensal, because the *Porcellio scaber* intestine turned out to be a suitable environment for *Bt* development. Margulis [125] believes that *Bt* not only colonizes the digestive tract of terrestrial crustaceans, but also establishes a symbiotic relationship with the host. Both authors observed the filamentous growth of bacteria, which probably caused incomplete cell fission and sporulation deceleration in the hypoxic environment of the host gut.

*Bt*-based pesticides are usually expected to be highly specific and eliminate only target insect species. Apart from the herbivorous insects, which constitute the majority of agricultural pests, entomophagous insects and pollinators may become exposed to *Bt* or its toxins, as a result of pesticide application. Entomophages play a vital role in the ecological balance and help control the number of pests at levels acceptable for agriculture. *Bt* is considered to be innocuous for entomophages due to the specific ligand-receptor mode of action of its major toxins [126]. In theory, *Bt* may infect non-target insects by direct exposure or through body surface ruptures; however, usually, the infection occurs when the non-target animal feeds on or lays its eggs in the infected herbivore. Bioaccumulation of Cry1Ac and Cry1F toxins was demonstrated in ladybird predator *Harmonia axyridis* fed with treated aphid *Myzus persicae* [127]. *Bt*- toxins were detected both in pupae and in unfed adults in a concentration higher than in their aphid prey. The authors argue that bioaccumulation secures a prolonged persistence of Cry toxins in the food web, allowing further contagion, for example, through cannibalism, interspecies predation, and exposure to parasitoids.

The safety of Cry1Ac, Cry1Ab, and Cry2Ab toxins has been shown for green lacewing *Chrysoperla carnea* when fed with the treated *Helicoverpa armigera* larvae, and for Cry1Ab and Cry1Ac similar results were obtained upon direct exposure [128,129]. The observed safety may be attributed to the apparent lack of Cry-binding receptors in the lacewing midgut epithelium. However, Hilbeck [130] demonstrated that CrylAb toxicity to *C. carnea*, with immature mortality was 57%, against the 30% observed in the control group. Negative effects for *C. carnea* were also reported upon its feeding with *S. littoralis* larvae exposed to a lethal concentration of Cry1Ab, although the observed effects might be mediated by the diminished nutritional value of intoxicated larvae, rather than by direct toxicity [131]. When conducting experiments implying the predator’s exposure to Cry toxins through the intoxicated prey, one should bear in mind that the obtained results might be mediated by the lowered nutritional value of such prey, and that in natural conditions, these effects might appear negligible given the possibility of the predator to choose from both infected and unaffected prey.

For predatory bugs, the data on the *Bt* toxicity differ depending on the experimental setup and the *Bt* strains involved. For *Podisus nigrispinus*, the exposure to the *Bt* var *aizawai* strain GC-91 contained in the prey and solution negatively affected the phytophagy, reproductive capacity, and biological cycle [132]. On the contrary, when fed with *Plutella xylostella* larvae treated with the *Bt* var. *kurstaki* and *Bt* var. *aizawai* CG 91, *P. nigrispinus* demonstrated an increase in predation activity [133]. Finally, *P. nigrispinus* demonstrated no preference between intact *P. xylostella* larvae and those treated with *Bt* HD1, and consumption of the latter did not affect the biological characteristics of the predator [134]. Since no receptors for Cry toxins were found in the midgut of this predator [135], any negative effects may be attributed to a decrease in the nutritional qualities of the infected prey. Side-effects of beta-exotoxin (thuringiensin), as well as the non-exotoxin-containing strain of *Bt* var. *kurstaki*, were demonstrated for pirate bugs *Orius albidepennis* [136]. The predator feeding on treated *Agrotis ypsilon* demonstrated a reduction in egg production, change in terms of nymphal duration and food consumption [136].

Similar to entomophagous insects, predatory spiders also contribute to the control of insect pests in agriculture. The prey range of spiders includes herbivorous insects, as well as species of higher trophic levels [137]. *Bt* affects the key metabolic enzymes, acetylcholine esterase (AchE), glutathione peroxidase (GSH-Px), and superoxide dismutase (SOD) of spiders. When *Ummeliata insecticeps* and *Pardosa pseudoannulata* were feeding the Cry1Ab-treated *Drosophila* flies, the expression of the respective genes decreased [138]. It is apparent that *Bt* affects the normal functioning of the nervous system of predatory spiders, including behavioral functions such as reproductive behavior, through the influence on the activity of these enzymes.

Parasitoids may be exposed to *Bt* when parasitizing in insect pests. A larval parasitoid fly, *Exorista larvarum,* did not demonstrate any decrease in the parasitism rate, puparium weight, or the number of progeny, when parasitizing on the *Bt*-treated *G. mellonella* host [139]. However, the negative effects of *Bt* were shown in a similar experiment for a parasitoid wasp *Palmistichus elaeisis* [140]. The concentration of *Bt* in the host larvae *S. frugiperda*, both resistant and sensitive, affected the number of larvae and pupae of *P. elaeisis*. At the same time, 15 days after parasitism, the number of parasitoid larvae in *Bt*-susceptible *S. frugiperda* was lower than in *Bt*-resistant *S. frugiperda* [140]. Similar results were reported for immature wasps *Trichogramma bourarachae* [141] and *Diadegma insulare* [142]. The insecticidal action of *Bt* affects insect populations’ growth rate, longevity, reproduction, and survivorship at different stages, compromising parasitoid larvae and their progeny. The parasitoid *Tranosema rostrale rostrale* did not exhibit preference to the *Bt*-treated prey [143]. The presence of commercial *Bt*-based formulations (Agree^®^ and Dipel^®^) and native *Bt* strains (HD1 and HD11) on *Helicoverpa zea* eggs did not affect the parasitism, larval survival, or adult emergence of *Trichogramma pretiosum* [144].

Possible effects of *Bt* on pollinator species deserve special consideration. Honeybees, one of the most successful pollinators on Earth, frequently serve as a test object in the assessment of novel insecticides’ off-target effects. According to the Guide to Biocontrol Agents (2014), *Bt*-based pesticides are safe for bees if their LD50 is lower than 0.1 mg/bee [37]. The pronounced negative effect on bees may be expected primarily for preparations containing exotoxin or exotoxin-producing strains [145]. However, Dipel^®^ commercial formulation, which does not contain exotoxin, caused the death of newly emerged adult *Apis mellifera* honeybees when mixed in pollen at a high concentration (1%) [145]. In low concentrations (0.25%). Dipel^®^ did not affect the rate of food consumption and survival, and neither did Foray^®^ 48B based on *Bt* var. *kurstaki* (0.25%), and purified Cry 1Ba δ-endotoxin from *Bt* strain Bt4412 (1, 0.25 and 0.025% *w*/*w*) [145]. Histological changes in the epithelium of *Apis mellifera* were shown when feeding candy paste with spores of *Bt* strains IPS82, BR81, and BR147, added at concentrations of 3.0 × 10^8^ spores/mL [146]. The purified and activated Cry1Ah toxin of *Bt* strain Bt8 did not demonstrate any negative effect on the survival, pollen consumption, and hypopharyngeal gland mass of honeybees *A. mellifera* and *A. cerana* when testing at 10 μg/mL, 10 ng/mL, and 1 ng/mL concentrations [147]. Two commercial preparations based on the *Bt* var. *kurstaki* (Dipel^®^) and *Bt* var. *aizawai* (Xentari^®^) were safe for bumblebee *Bombus terrestris* when exposed dermally or by feeding the treated pollen at 0.1% concentration [148]. However, when feeding bumblebees with sugar water with 0.1% of preparations, *Bt* var. *aizawai* caused the total mortality of working bumblebees and reduced reproduction by 100%. No lethal effect was observed with a decrease in concentration [148]. As for lepidopteran pollinators, the current data on the off-target *Bt* activity is practically absent. Moths are the most important nocturnal pollinators of various plants in many ecosystems [149]. It should be noted, however, that the larval stages of several important pollinators represent herbivorous pests. Although adult insects are not sensitive to *Bt* infection, the eradication of their larval stages may have a long-term effect on the plants pollinated by lepidopterans.

All of this considered, the negative effects of *Bt* preparations on non-target invertebrates appear to be multidirectional. First, *Bt* may exert a direct toxic effect on these organisms, either with its Cry toxins or via its repertoire of non-selective virulence factors. Second, *Bt* can alleviate the sustenance of entomophages and parasitoids by decreasing the quantity of their prey or hosts, respectively, or by debilitating the prey’s nutritional value (Figure 1). In turn, non-target invertebrates may act as a reservoir for the bacterium, participating in its dissemination. In such organisms as invertebrates feeding on roots (Nematoda), soil organic matter (earthworms), vegetal debris (Isopoda), and leaves, *Bt* may act as either commensal or pathogen. Since invertebrates constitute a substantial part of vertebrates’ prey base, they may eventually serve as vectors transmitting *Bt* to vertebrate insectivores.

## 5. Vertebrates

Large vertebrates represent the higher trophic levels of practically all food chains in terrestrial ecosystems. Although these organisms are considered to be suboptimal hosts for *Bt* propagation, any possible adverse effects of *Bt* on vertebrates should be studied thoroughly to secure its safety in agricultural practice. To this end, a lot of data have been accumulated regarding the safety of *Bt*-based pesticides for mice, rabbits, fish, poultry, pigs, calves, cattle [150,151,152], and human volunteers [153].

Non-target aquatic vertebrates can be exposed to *Bt* as a result of biological treatments aimed against insect pests or mosquitos. Most researchers report that *Bt* spores parasporal bodies are not toxic for fishes [152,154,155,156,157]. Snarski [157] observed no adverse effects of exposure of *Bt* var. *israelensis* (*Bti*) on fathead minnows, *Pimephales promelasandas*, or fish mortality. Moreover, upon the transfer to *Bt*-free water, fishes effectively excreted the toxins from their organisms [157,158]. No genotoxic or mutagenic effects of *Bt* toxins on peripheral erythrocyte cells of *Oreochromis niloticus* were observed [154]. Cry1Ba6 and Cry10Aa toxins did not show adverse effects to *O*. *niloticus,* although Cry1Ia induced slight DNA damage when applied at extraordinarily high concentrations [154]. In order to provide a more precise assessment of *Bt* toxicity to fishes, several studies utilized hematological parameters as indicators of total fitness. For instance, the intra-abdominal *Bt* injection (0.2 mL of 1 × 108 spores/mL) caused the increased apoptosis rates in *O. niloticus* erythrocytes, although no acute toxicity was observed when fishes were placed in an aquarium with *Bt* solution for 72 h [151]. Short-term *Bt* presence both in the surrounding water and diet of *Piaractus mesopotamicus* altered blood properties and slightly reduced fish appetite without, however, elevated mortality rates [159].

The effects of *Bt* on amphibians appear to be similar to those observed in fishes. No increase in mortality was observed in *Rana temporaria* tadpoles after applying preparations based on *Bti* [160,161]. However, tadpoles treated with *Bti* demonstrated faster development compared to untreated controls. Besides, the use of *Bti* induced significant increases of glutathione-S-transferase (37–550%), glutathione reductase (5–140%), and acetylcholine esterase (38–137%), which may indicate detoxification, antioxidant reactions, and a change in the activity of neurons [161]. Most other studies have shown no toxic effect of *Bti* on amphibians (larval frogs, newts, salamanders, and toads) [160,162]. An exception, however, is *Leptodactylus latrans* tadpoles, which demonstrated 100% mortality when treated with Introban^®^, a pesticide based on *Bti*, at a concentration of 40 mg/L, while sublethal concentrations altered the oxidative stress enzymes’ activity and caused genotoxicity and intestinal damage [163]; however, it is unclear whether the observed effects were caused by *Bti* itself or by other components of the formulation.

In mammals, *Bt* may reside in intestinal organs with no visible signs of infection and thus be found in rectal or fecal probes of apparently healthy specimens [60,61]. However, depending on its virulence factor repertoire and the immune status of the host, *Bt* may behave as an opportunistic pathogen [164]. For instance, mice inoculated intranasally with 10^2^, 10^4^, and 10^7^ spores of *Bt* strain H34 did not exhibit any mortality, while the combined infection with the same doses of *Bt* and 4% of the lethal dose of influenza A virus killed 40%, 55%, and 100% of mice, respectively [165]. In humans, the bacterium identified as *Bacillus thuringiensis* subsp. *konkukian* (Serotype H34) was isolated from wound abscess of an immunocompetent person in 1995 [166]. To prove the *bona fide* pathogenic status of the isolated bacterium. Its suspension cultures were applied to the depilated areas of the skin of laboratory mice differing in their immune status at concentrations of 10^5^, 10^6^, or 10^7^ CFU. Although skin inflammation occurred in all groups when exposed to 10^7^ CFU inoculum, the regeneration rates were significantly lower in the immunosuppressed specimen, and necrosis was observed more frequently in this group [166].

The pathogenic properties of *Bt* described above may be caused by any of the minor virulence factors produced by *Bt* [15]. Since most of these factors demonstrate low host specificity, their presence in a certain strain may condition its pathogenicity to humans and other vertebrates. This is especially true for low weight toxic moieties, such as β-exotoxin, which was demonstrated to exhibit adverse effects on mammalian cells [167,168,169,170]. Furthermore, *Bt* strains frequently bear genes encoding for hemolysins, which define pathogenetic traits in *B. cereus*. Hemolysins detected in *Bt* genomes relate to types I [171], II [172], and IV [173]; for the latter, the frequency of their presence was estimated as 84%, based on an assay containing 205 strains. In a similar study, all of 74 *Bt* strains tested harbored genes encoding for *B. cereus* enterotoxins, and all but one managed to inhibit protein synthesis in Vero cells, verging the toxicity values of *bona fide B. cereus* strains [174].

Possible risks of *Bt* use may be associated with the genetic material exchange between bacteria of the *Bc* group [175,176,177]. Transfer of the insecticidal plasmid pHT73 by means of conjugation has been shown from *Bt* to *B. cereus*, *B. mycoides,* and even to *B. anthracis,* the etiological agent of anthrax [103,178,179]. In the respective studies, the recipient strains produced the crystalline Cry1Ac protein encoded by the plasmid pHT73, which demonstrated positive toxicity to *Helicoverpa armigera* larvae. The ability of plasmid transfer from an emetic *B. cereus* strain to *B. thuringiensis* was demonstrated in milk-borne communities [177]. A similar exchange of genetic material may also occur between the representatives of different *Bt* serovars [175]. At the same time, a recent study demonstrated that sequences similar to the pXO2 plasmid from *B. anthracis* are also present in some *Bt* and *B. cereus* genomes [178]. In this light, previously accepted functional species signatures, such as pXO plasmids in *B. anthracis* and Cry gene plasmids in *B. thuringiensis*, may not be restricted to these species [11]. The potential for causing specific diseases is also not species-constrained since the new evidence shows that specific *B. cereus* strains varying in their genotypes and phenotypes may cause anthrax-like disease [179]. Given the possible outcomes that gene exchange with human pathogens may have in *Bt*, special attention should be paid to genetic control of the strains used for biocontrol and their potential for horizontal gene transfer.

The study of *Bt* toxicity for humans has a long history that includes testing on volunteers [153]. One of the most large-scale studies of this kind regarded the isolation of *Bt* var. *kurstaki* from people living near areas of air dispersion of an insecticide based on it [180]. 55 *Bt* isolates were found, 95% of which did not cause any health problems in humans. However, for three patients with health problems in medical history, *Bt* was proposed as a probable causative agent of their diseases. The concerns that *Bt* may adversely affect human health upon constant exposure to pesticides and preceding immunity weakening have been articulated. According to several reports, *Bt* infection occurred in immunocompromised people, but the loss of experimental data deteriorates their credibility [150].

The possible involvement of *Bt* in food-borne outbreaks was noted in two studies. *Bt* isolates were found in the stool of patients suffering from a gastroenteritis outbreak alongside with *B. cereus* strains [181]. All *Bt* isolates showed cytotoxic effects characteristic of enterotoxin-producing *B. cereus.* However, this does not indicate the association of *Bt* with the disease; besides, in some patients, Norovirus was also detected [181]. *Bacillus cereus* group bacteria were also identified in samples collected from food and clinical samples during food poisoning outbreaks from 1991 to 2005 in Canada [182]. *B. cereus* was detected in most samples, while *Bt* was identified in four food samples exclusively. A more accurate systematic position of bacteria was not determined, and there are no data on whether *Bt* found originate from the preparations usage. A similar foodborne outbreak was also recorded in 2012 in Germany, in which three individuals consumed a salad containing *Bc* group bacteria identified as *Bt* [47]. Perhaps undetected *B. cereus* was also contained in lettuce and had a synergistic effect with *B. thuringiensis,* causing the symptoms of the disease [174].

It is apparent that *Bt* rarely exerts direct negative effects on vertebrates. In most of the cases, the observed clinical pictures can be at least partly attributed to the minor components of the pesticidal preparations, high concentrations of pesticide applied, concomitant diseases. Few remaining examples involve either the presence of closely related *Bc* group species or deal with the recently evolved strains having acquired novel virulence factors. Although spuriously consumed *Bt* spores are not completely digested in the gastrointestinal tract of vertebrate animals, they do not advance further to blood and other tissues of the host organism and thus can be perceived harmless to it. Nonetheless, *Bt* still may have some indirect effects on vertebrates, such as alterations in food sources reducing its abundance and nutritional value, which are best tracked down if the whole complexity of relationships occurring in the natural ecotopes is considered.

## 6. Land Ecosystems

The direct impact of pesticides on species at one trophic level can further reverberate throughout the trophic chains [183]. In this regard, a thorough assessment of *Bt*’s impact on natural habitats should consider not only the organisms vulnerable to direct exposure but also those they are connected with through trophic relationships.

The use of *Bt* var. *israelensis* (*Bti*) as a mosquito control agent detriments higher trophic levels, mainly by reducing food sources, ultimately affecting vertebrate predators. In one assay, common house martin *Delichon urbicum* changed food preferences in the treated areas, switching from midges and spiders to flying ants [184], which affected the adult fecundity and survival of the chicks [184]. Preparations based on *Bt* var. *kurstaki* can also affect vertebrates by reducing the number of lepidopteran larvae, an important food source for some birds and bats [185]. The secondary effects of *Bt* var. *kurstaki* were shown for spruce grouse (*Dendragapus canadensis*) [186], whose chicks suffered from 30% body mass loss, due to the lack of lepidopteran larvae in their diet in the exposed areas. Conversely, the study aimed at evaluation of the lepidopteran pest treatment outcomes for Tennessee warblers *Vermivora peregrina* showed no significant differences in the number of chicks and their survival. Black-throated blue warblers (*Dendroica caerulescens*) prefer to avoid nesting in the *Bt*-treated territories, but the overall clutch size, hatching rate, and fledgling number were not reduced in the studied populations [187].

Pesticides based on *Bt kurstaki* are used to control the gypsy moth *Lymantria dispar* in the forest ecosystem. Four-year application led to changes in food supply behavior of adult worm-eating warblers (*Helmitheros vermivorus*), increasing the time they spent for food search, which resulted in a decrease in nestlings’ body mass [188]. On the other hand, *Bt* var. *kurstaki*-mediated *L. dispar* control did not affect the diversity of songbird species or the number of chicks in each species with the exception of *Pipilo maculatus,* for which a significant decrease in the offspring number was shown [189].

Raimondo and colleagues conducted a two-year study of the connection between *Bt* var. *kurstaki* treatments and the salamander’s dietary changes [190]. In food chains, salamanders may link insects to predatory birds and mammals. Although the diet of five species of the Plethodontidae family (*Desmognathus fuscus, D. monticola, D. ochrophaeus, Plethodon cinereus, P. glutinosus*) partially consisted of lepidopteran larvae eradicated with biopesticides, the researchers did not notice any change in the number of salamanders feeding on the treated areas, nor did they note the difference in the proportions of the major prey species in their diets [190].

As its major side effect, reducing the number of lepidopterans can be critical to the survival of rare species feeding predominantly on caterpillars. Among the endangered bat species that may be threatened this way are Virginia big-eared bat (*Corynorhinus townsendii virginianus*) and the Indiana bat (*Myotis sodalist*) [191,192]. In addition, rare plant species depending on the lepidopteran pollinators of a certain family may be under the threat of extinction. For example, spraying *Bt.* var. *kurstaki* can affect pollinators of the family Sphingidae, which subsequently affects Eastern prairie fringed orchid (*Platanthera leucophaea*) [192].

Judging by the data gathered, the effects of *Bt*-based preparations dissected at the ecosystemic level confines mostly to the reduction in the quality and quantity of the insectivores’ food supplies. Such influence poses an utter danger to species with narrow dietary preferences. Moreover, even in species with diverse food bases, nutritional deficiency may negatively affect the younglings, who are more vulnerable to dietary switches and insufficiency (Figure 1). Thus, animals tolerant to the direct exposure to *Bt* can be negatively affected by pesticides’ application in the long run.

## 7. Aquatic Ecosystems

Apart from terrestrial communities, indirect changes at the ecosystemic level caused by *Bt* introduction can be observed by *Bt*’s presence in aquatic environments. *Bt* mostly reaches for such habitats as a component of mosquito control pesticides’ application, predominantly *Bti* spores and toxins [193]. Apart from that, *Bti* can be detected in natural environments not exposed to treatment, along with other serovars (such as *kurstaki, mexicanensis, sumiyoshiensis, seoulensis*) less commonly found in water [194]. Notably, the insecticidal activity of the *Bti* preparations is maintained for more than 5 months coupled with spore recycling regardless of the presence [70] or absence [195] of mosquito larvae, as well as in the specific breeding sites containing leaf litters [74]. Spores and toxins may become encapsulated within the protozoan *Tetrahymena pyriformis* cells, wherein the spores were demonstrated to germinate inside the vacuoles and with further filamentous cell growth [196]. Thus, *Bt* can potentially preserve and recycle in organisms unrelated to target hosts. Although *Bti* is considered non-toxic to most aquatic non-target organisms [193,195], its toxicity for zooplanktonic microcrustaceans that coexist with target mosquito larvae [197] and to non-biting midges (Chironomidae) [198] was reported.

Chironomids, the non-biting mosquitoes, are considered a major component of the wetland food web [199]; therefore, considerable changes in their population can exert a far-reaching effect on the whole ecosystem [200]. When exposed to *Bti* in experiments conducted in both artificial mesocosms and actual field conditions, chironomid larvae decreased in their number by 53–87% [201]. A decrease in the number of chironomids, a priority food resource for dragonflies and newts, exacerbates food resources competition and respective intraguild predation. It was shown that the dragonfly *Aeshna cyanea* decreased the survival of newt larvae by 27% under chironomids shortage after *Bti* treatments [201]. Lundström et al. [202] did not report any *Bti*-induced deterioration for mosquito control, thus claiming that *Bti* usage is safe for birds, bats, or any other predators feeding on chironomids. Additionally, the absence of a direct or long-term detectable effect of VectoBac^®^ WDG and 12AS preparations on the taxonomic structure and taxon population of non-target communities of aquatic invertebrates, including chironomids, has been declared [203]. Nonetheless, Jakob and Poulin [199] showed the negative consequences of *Bti* usage for dragonflies and damselflies (Odonata) preying on mosquitoes and chironomids and being, in their turn, consumed by birds. The abundance and species diversity of Odonata were significantly higher in the control areas compared to the *Bti*-treated areas. In addition, in the treated areas of the same region, a 33% decrease in the survival rate of swallow chicks was observed due to a decrease in the proportion of Odonata and Chironomidae in the diet [184].

Apparently, *Bti* can negatively affect the number of chironomid larvae with regular treatments with mosquito repellents [199,201]; however, the negative effect of bacteria on chironomids has not been universally acclaimed [202,203,204,205]. Nevertheless, the change in the availability of chironomids as a food resource can affect the reproductive success of birds [184,206] and the species diversity of dragonflies [199]. The reduction in the chironomid population altered prey preferences and induced intraguild predation between dragonflies and newts (Figure 1) [201]. One can speculate whether similar alteration of trophic behavior can occur in other ecosystems or between species indiscriminate in their size and predatory competencies within their ecological guild.

In addition to pesticides based on *Bti*, which are used to control mosquitoes directly in water areas, aquatic non-target organisms may experience a negative effect from *Bt* used overland and accidentally reaching the water. It was proven that contamination of watersheds with *Bt* var. *kurstaki* is unlikely to directly affect aquatic insects, including various species of Ephemeroptera, Plecoptera, and Trichoptera orders [207,208,209]. The authors did not detect any significant mortality, drift response, or changes in the taxonomic richness of benthic invertebrates after the application of *Bt* var. *kurstaki*. Therefore, *Bti* remains the only known serovar for which the detectable effects on the aquatic ecosystems have been described.

## 8. Conclusions

Although exact environmental consequences of intensive biopesticide usage are yet to be investigated, the data available suffice to provide a first glimpse into a network of complex relationships involving *Bt* in diverse ecosystems and ecotopes (Figure 1). However, the long-term coexistence and coevolution of *Bt* with different taxa hinders any unambiguous judgment on the nature of their mutual influence. The only obvious thing is that because it is a natural component of ecosystems, *Bt* can interact directly or indirectly with different groups of organisms, from bacteria (including horizontal gene transfer, which is out of the scope of the present review) to mammals and human beings. Such a complex and tightly balanced network of interactions might undergo drastic and unpredictable alterations upon the extensive introduction of new *Bt* strains. Putting it simply, there is no simple answer for the ‘to spray or not to spray’ question. Even with the widely acclaimed safety of Bt-based pesticides and the benefits they offer notwithstanding, any introduction of alien bacteria to the ecosystems eventually triggers consequences outside the targeted pest species, and unconstrained overuse of biopesticides produces effects that might be both inconspicuous and detrimental for the environment. To escape this, the long-reach and long-term consequences of the *Bt* introduction into nature should be considered, and specific strains and corresponding preparations should be constructed to narrow down possible side-effects.

## Data Availability

Not applicable.
